# Novel Interactions of Myristic Acid and *FADS3* Variants Predict Atopic Dermatitis among Indonesian Infants

**DOI:** 10.3390/nu14214676

**Published:** 2022-11-04

**Authors:** Conny Tanjung, Carla P. Harris, Hans Demmelmair, Sarah Dwitya, Zakiudin Munasir, Herawati Sudoyo, Marie Standl, Damayanti Rusli Sjarif, Berthold Koletzko

**Affiliations:** 1 Graduate School, Hasanuddin University, Makassar 90245, Indonesia; 2 IMERI Faculty of Medicine, Human Genetic Research Cluster, Universitas Indonesia, Jakarta 10430, Indonesia; 3 Department of Pediatrics, Dr. von Hauner Children’s Hospital, LMU University Hospitals, LMU-Ludwig Maximilians Universität Munich, 80337 Munich, Germany; 4 Helmholtz Centre Munich-German Research Center for Environmental Health, Institute of Epidemiology, 85764 Neuherberg, Germany; 5 Kemayoran Public District Hospital, Central Jakarta, Jakarta 10650, Indonesia; 6 Departments of Pediatrics, Faculty of Medicine, Universitas Indonesia, Dr. Ciptomangunkusumo National Referral Hospital, Jakarta 10430, Indonesia; 7 Eijkman Institute for Molecular Biology, Jakarta 10430, Indonesia

**Keywords:** atopic dermatitis, fatty acids, *FADS* gene variants, metabolism, infants

## Abstract

Fatty acids exert a range of different biological activities that could be relevant in the development of atopic dermatitis (AD). This study investigated the association of glycerophospholipid fatty acids (GPL-FA) with AD, and their interactions with single nucleotide polymorphisms (SNP) of the *FADS1-3* gene cluster. Among 390 infants of the Indonesian ISADI study, GPL-FA were measured in umbilical plasma (P-0y) and in buccal cells at birth (B-0y), and again in buccal cells at AD onset or one year (B-1y). Prospective and cross-sectional associations with AD were assessed by logistic regression. Interactions of GPL-FA with 14 SNP were tested assuming an additive model. AD was diagnosed in 15.4% of participants. In B-1y, C18:2n-6 was inversely associated with AD; and positive associations were observed for C18:1n-9, C20:4n-6, C22:6n-3 and C20:4n-6/C18:2n-6. There were no prospective associations with AD, however, a significant interaction between the SNP rs174449 and B-0y C14:0 (myristic acid) was observed. This study indicates that Indonesian infants with AD have increased rates of endogenous long-chain polyunsaturated fatty acid production, as well as higher C18:1n-9 levels. GPL-FA measured at birth do not predict later AD incidence; however, genotype interactions reveal novel effects of myristic acid, which are modified by a *FADS3* variant.

## 1. Introduction

Atopic dermatitis (AD) is one of the most common inflammatory diseases among infants, with about 60% of cases manifesting in the first year of life [1]. The disease is characterized by itchy and inflammatory skin lesions, and is associated with a number of comorbidities, such as digestive problems, ocular complications, and autoimmune diseases [2], having a substantial impact on patient physical and mental well-being [3,4]. Patients with AD can be more susceptible to bacterial and viral infections [5], an aspect of immense relevance in the midst of the COVID-19 pandemic. Indeed, there have been reports of increased odds of COVID-19 infection among AD patients, irrespective of other comorbidities [6]. An exacerbation of AD symptoms has also been noted, presumably linked to the strict hygiene measures and increased stress levels imposed by the pandemic [7]. Furthermore, in what has become widely known as the atopic march, AD often precedes the onset of allergic rhinitis and asthma, which typically occur later in childhood [8]. Primary prevention of AD could hence extend far beyond the condition itself, reducing the risk of developing other diseases and long-term health problems, improving quality of life and reducing the burden on health-care resources. Nevertheless, despite much valuable research contributing to this goal, the search for more effective strategies continues [9,10].

Fatty acids are involved in a range of different biological activities, including intracellular signaling pathways, gene expression, and the production of bioactive lipid mediators [11]. As constituents of phospholipids, they are required for cell membrane structure [12], and are fundamental components of the skin [13]. Indeed, differences in skin lipid composition have been observed in relation to AD [14]. While the relevance of saturated, monounsaturated, and trans fatty acids has been widely discussed in other disease contexts [11], the vast majority of AD research focuses on the roles of omega-3 and omega-6 long-chain polyunsaturated fatty acids (LC-PUFA), since these fatty acids are specifically involved in regulating inflammation [15]. Potent pro-inflammatory lipid mediators are derived from the omega-6 arachidonic acid (ARA; 20:4n-6), whereas mediators derived from omega-3 eicosapentaenoic acid (EPA; 20:5n-3) and docosahexaenoic acid (DHA; 22:6n-3) exert anti-inflammatory and inflammation-resolving functions [16,17]. Evidence regarding the impact of early LC-PUFA exposure on infant AD is largely inconsistent [15]. This could partly be related to intra-individual differences in the rates of conversion of dietary PUFA to LC-PUFA, which can be influenced by gene variants in the coding genes of fatty acid desaturase enzymes (*FADS* genes) [18]. Gene–diet interaction studies have indeed demonstrated that *FADS* gene variants can modulate the association between dietary fatty acids and allergic diseases [19,20].

An important finding among Indonesian participants of the ISADI (Indonesian Prospective Study of Atopic Dermatitis in Infants) birth cohort, was that in contrast to Europeans, alleles predicting a fast conversion of dietary PUFA to LC-PUFA are only carried by a minority of the population [21]. Thus, one cannot meaningfully extrapolate results obtained from observational and intervention studies in European to Indonesian populations. Additionally, with respect to research in AD, it is known that a variety of endotypes exist, also presenting marked geographical differences that need to be considered separately [22].

To our knowledge, no study has yet assessed infant fatty acids and their interactions with *FADS* gene variants in relation to AD incidence in a South East Asian population. The ISADI study was specifically designed with this intention, comprising a large sample of infants with several genotyped variants of the *FADS1-3* gene cluster, and fatty acids measured in buccal cheek cells collected at birth and again at the time of AD diagnosis or at 1 year of age. Since blood collection is not always readily accepted among infants or their parents, the analysis of fatty acids in buccal cells offered a valuable alternative within the present study setting [23].

## 2. Materials and Methods

### 2.1. Study Population

The study involved 390 infants enrolled in the ISADI study, conducted at the Kemayoran Primary Health Care, central Jakarta, from April 2014 to December 2015. The primary endpoint of the ISADI study is to assess the role of *FADS1-3* gene polymorphisms and LC-PUFA composition on the incidence of atopic dermatitis. The study protocol has been described in detail elsewhere [24]. Briefly, apparently healthy newborn infants (37–42 weeks of gestation) with a birth weight ≥2500 g, whose mothers did not supplement with omega-3 or -6 PUFA, were included.

### 2.2. Data Collection

Physical examinations were performed every 3 months to assess AD, diagnosed by Hanifin-Rajka criteria. Information on relevant covariates was collected by questionnaires. From birth up to 12 months, monthly monitoring by telephone was carried out to record breastfeeding and complementary feeding practices. The present study was conducted according to the International Ethical Guidelines for Biomedical Research Involving Human Subjects. All procedures were approved by the Permanent Medical Research Ethics Committee in Medicine and Health/Faculty of Medicine Universitas Indonesia/Dr Cipto Mangunkusumo Hospital (47/H2.F1/ETIK/2014, extended by letter 148/UN2.F1/ETIK/2015). Written informed consent was obtained from the parents of all study subjects. The study was registered at clinicaltrials.gov as NCT02401178.

#### 2.2.1. Sample Collection and Handling

Umbilical artery plasma (P-0y) and buccal cheek cells (B-0y) were sampled at birth. A second buccal cheek specimen was obtained at AD diagnosis or at age 1 year (B-1y). Umbilical artery plasma was collected into EDTA tubes directly after birth before cord clamping. Specimens were immediately centrifuged. The plasma and buffy coat were frozen at −80 °C. Buccal cheek cells were collected within 1 h of birth by brushing the surfaces of the inner mouth mucosa 20–25 times with gentle pressure, using a Gynobrush (Herenz 1032929, Heinz Herenz Medizinalbedarf, Hamburg, Germany). The brush was then put into a Sarstedt tube (62.554.502, 15 mL) and held in place by the tube cap, so that the cells stuck to the brush made sedimentation after centrifugation, as described in patent EUPCT/EP2011/003829. Centrifugation was performed at 1400× *g* for 10 min at 4 °C and the supernatant removed. The same procedure was followed for the second sample of buccal cells. All specimens were immediately frozen at −80 °C and transported on dry ice by air to LMU Munich, Germany, for the analysis of GPL-FA.

#### 2.2.2. GPL-FA Measurements

Umbilical plasma and buccal cheek cell GPL-FA were quantified as described previously [21,23]. Briefly, selective preparation of methyl ester derivatives of GPL-FA was achieved by coprecipitation of triacylglycerols and cholesterol esters with proteins and base-catalyzed transesterification excluding methyl ester synthesis from nonesterified fatty acids. Aliquots (100 μL) of EDTA plasma were combined with methanol containing dipentadecanoyl phosphatidylcholine as an internal standard. Tubes were centrifuged (3030× *g*; 10 min; 4 °C) and the supernatant transferred to another vial. Sodium methoxide solution was added for fatty acid methyl ester (FAME) synthesis from GPL-FA at room temperature. After 4 min, the reaction was stopped with methanolic HCl, and the FAME was extracted into 1 mL hexane for gas chromatographic analysis. The FAME was quantified by gas chromatography with flame ionization detection (Agilent 7890 GC; Agilent, Waldbronn, Germany), using a 50-m, 0.22-mm inner diameter BPX70 column (SGE, Weiterstadt, Germany). Injection was performed with a programmable temperature vaporizer (Gerstel, Mühlheim, Germany) to avoid preconcentration of hexane extracts before gas chromatography. FAME peaks were identified and calibrated relative to pentadecanoic acid methyl ester (internal standard) by comparison with a FAME standard mixture (GLC-569B; Nu- Check Prep, Inc., Elysian, MN, USA). A total of 27 GPL-FA were measured and are reported as a percentage of total GPL-FA (wt%) [23].

#### 2.2.3. Genotyping

Genotyping was performed at the Research Unit of Molecular Epidemiology at Helmholtz Zentrum Munich, Germany, as previously described [25]. DNA was extracted from the buffy coat of umbilical artery blood by the Puregene DNA isolation kit (Gentra Systems, Hilden, Germany). Genotyping was performed using the iPLEX Gold Chemistry (Sequenom, Hamburg, Germany) and matrix-assisted laser desorption ionization-time of flight mass spectrometry, with methods to detect allelic differences. In brief, locations containing certain SNPs were amplified by polymerase chain reaction, using specific primers. After deactivation by alkaline phosphatase, single-base elongation was performed in accordance to the print order. After salt ion removal by ion switch and elongation reaction, the specimen was transferred to a silicone chip and covered with 3-hydroxypicolinic acid. The differences from specific alleles were measured by the matrix-assisted laser desorption ionization-time of flight mass spectrometry. Allele recognition from SNPs was performed by Mass ARRAY Typer version 4.0.5 (Sequenom, Hamburg, Germany). SNPs for FADS genes were selected based on 3 criteria: (1) the SNP has been studied in previous publications; (2) the SNP candidates being considered are SNPs that have already been demonstrated to be associated with LC-PUFA status; and (3) minor allele frequency (MAF) >10%.

#### 2.2.4. Statistical Analysis

All statistical analyses were performed using the statistical software R, version 3.6.2 [26]. Descriptive characteristics of the study population were summarized by counts (%) for categorical variables and by medians (interquartile range) for continuous variables, and presented for the total population as well as stratified by the presence/absence of AD. Differences in characteristics between subjects with and without AD were tested by Pearson’s chi-squared test and Wilcoxon Rank Sum test, for categorical and continuous variables, respectively.

Outliers in the levels of GPL-FA measured in P-0y, B-0y and B-1y were visually identified by means of boxplots, and these values were removed prior to further analyses. Levels of GPL-FA in each sample, and the relevant ratios thereof, were described by medians (interquartile range). Correlations between GPL-FA levels in paired samples were tested by Spearman’s *ρ*, i.e., between (a) P-0y and B-0y, (b) P-0y and B-1y, and (c) B-0y and B-1y. Associations between GPL-FA and AD were tested by logistic regression, adjusting for selected covariates (parental education [either mother or father graduated junior high or lower; graduated high school; diploma or above], sex [female; male], family history of atopy [yes; no], exclusive breastfeeding for at least 4 months [yes; no], paternal smoking [yes; no], number of siblings [0; 1; 2 or more], and frequency of illness in first 12 months of life [days]). Allele frequency, Hardy-Weinberg equilibrium (HWE) and linkage disequilibrium were assessed among the 18 tested SNP, using the R-package Genetics [27]. Genotypes were coded following an additive model (count of minor allele: homozygous major = 0, heterozygous = 1, homozygous minor = 2). To assess whether associations between GPL-FA and AD were modified by the selected *FADS* variants, interactions between each GPL-FA and each SNP were tested. In the case of a significant interaction, additional analyses were performed for the corresponding GPL-FA, stratified by the relevant SNP genotype. Statistical significance was defined using Bonferroni correction for multiple testing, dividing 0.05 by the number of GPL-FA analyzed (0.05/27 = 0.00185 in umbilical plasma, 0.05/21 = 0.00238 in buccal cells). When assessing the interaction effects, the alpha level was further divided by 11, which is the number of effective loci among the analyzed SNP computed according to Nyholt [28], using the meff function of the R-package poolr [29] (https://CRAN.R-project.org/package=poolr, accessed on 13 October 2022). Results are presented as odds ratio (OR) and 95% confidence interval (95%CI) for an interquartile range increase in the respective GPL-FA being tested.

## 3. Results

The study population included 390 subjects, in whom umbilical artery plasma (P-0y), and data on SNP genotype, AD and all selected covariates were collected. Descriptive characteristics for the total study population, subjects with AD, and subjects without AD, are summarized in Table 1. About half of the participants were female (49%). The majority came from families with a medium level of education (72%), were breastfed for at least 4 months (60%), had a father who smokes (76%), and no atopic family history (58%). About two thirds of subjects had one (36%) or at least two siblings (33%). Subjects were sick with cold, cough, or fever an average of 11 days in the first year of life.

Subjects with AD comprised 15.4 % of the population. These had a higher parental education compared to subjects without AD (15% vs. 7%), were more likely to have a family history of atopy (77% vs. 35%), and were more frequently sick (median of 24 days vs. 9 days).

The levels of GPL-FA (wt%) measured in each sample are shown in Table 2. Among the 390 participants with measured GPL-FA in P-0y, buccal cheek samples were collected in 387 participants at birth (B-0y) and a second buccal sample (B-1y) in 299 participants. Marked differences in GPL-FA levels were observed between samples, with greater C16:0, C20:3n-6, C20:4n-6, and C22:6n-3 levels in umbilical artery plasma than in either of the buccal cell samples. In contrast, C18:1n-9 was greater in buccal cells (B-0y and B-1y), and C18:2n-6 was higher only in B-1y samples.

### 3.1. Correlations between GPL-FA

**Figure 1:**
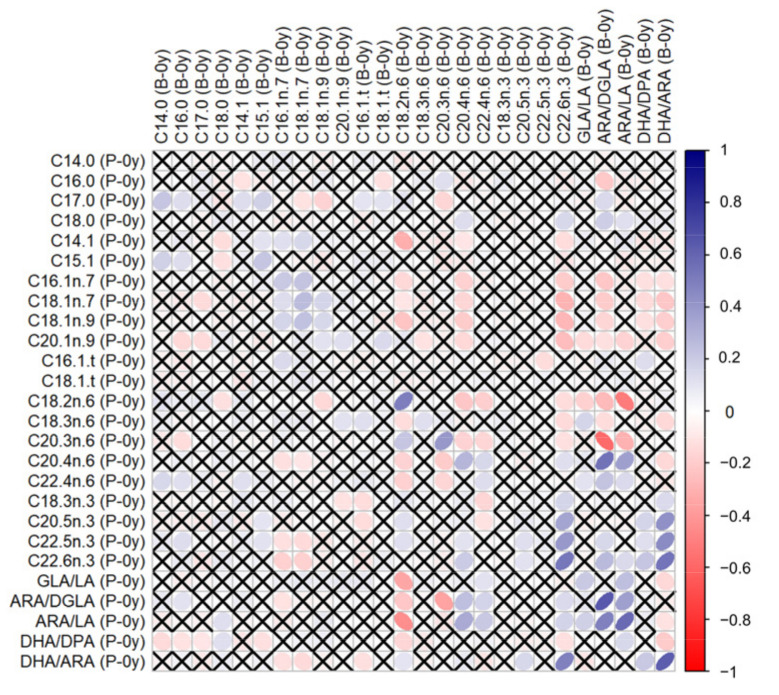
Correlations between GPL-FA levels measured in umbilical plasma (P-0y) and buccal cells at birth (B-0y). Tested by Spearman’s *ρ*. White-to-blue: increasing *ρ* (positive correlation); white-to-red: decreasing *ρ* (negative correlation). ✕ = no significant correlation.

Correlations between GPL-FAs measured in different samples can be found in the Supplementary Table S1. GPL-FAs measured in P-0y and B-0y presented stronger correlations with each other than when compared to measurements at follow-up (P-0y vs. B-1y or B-0y vs. B-1y). In particular, C18:2n-6, C22:6n-3, ARA/DGLA, ARA/LA and DHA/ARA in P-0y presented strong positive correlations (≥0.5) with their respective levels in B-0y, as illustrated in Figure 1.

### 3.2. Association of GPL-FA with AD

Results of logistic regression analyses are presented in Table 3. There were no significant prospective associations of GPL-FA measured at birth (P-0y or B-0y) with AD. In cross-sectional analyses assessing GPL-FA in buccal cells at follow-up (B-1y), a significant inverse association with AD was observed for C18:2n-6 (OR = 0.567 [95% CI = 0.397;0.800], *p* = 0.0015); and a positive association was observed with C18:1n-9 (3.539 [2.193;5.988], *p* < 0.001), C20:4n-6 (2.021 [1.358;3.072], *p* < 0.001), C22:6n-3 (2.032 [1.338;3.131], *p* < 0.001), and ARA/LA (1.832 [1.351;2.595], *p* < 0.001).

### 3.3. Effect Modification by FADS Variants

The minor allele frequencies (MAF) and HWE *p*-values of the 18 selected SNPs are presented in Table 4. One SNP (rs968567) was monomorphic and was not further analyzed. The other 17 SNPs were in HWE. The SNPs rs174548, rs174556, and rs174561 had an identical MAF of 26.7% and were highly collinear, and hence, only rs174548 was further analyzed. Similarly, rs174576 and rs174578 had an identical MAF of 21.6% and hence only rs174576 was further analyzed.

**Figure 2:**
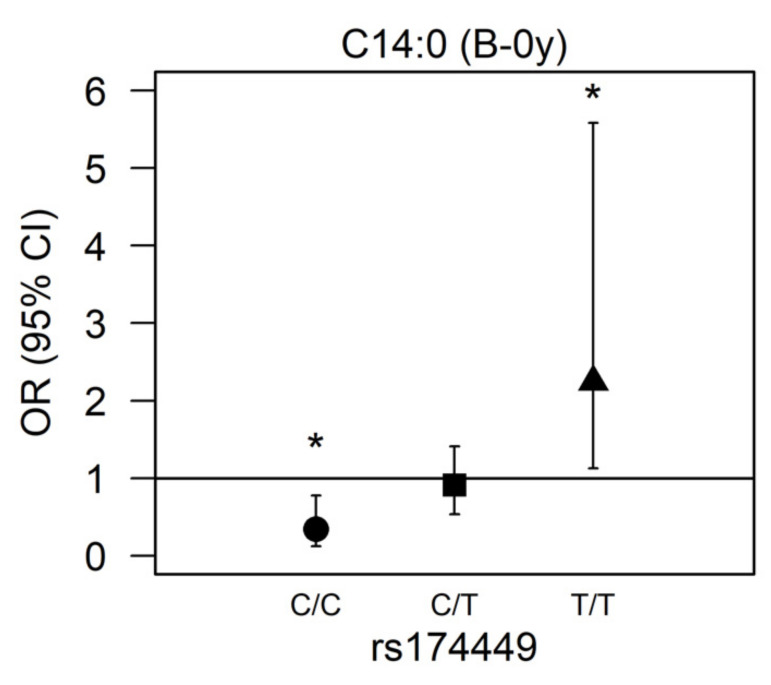
Association between myristic acid (C14:0) and AD modified by rs174449 genotype. B-0y: measured in buccal cells sampled at birth. * Nominal statistically significant (*p* < 0.05). ●: homozygous major genotype (C/C); ■: heterozygous genotype (C/T); ▲: homozygous minor genotype (T/T).

There was a significant interaction for rs174449 with C14:0 measured in buccal cells at birth (*p* = 0.00017). In subsequent analyses stratified by genotype, we observed nominally significant associations in both homozygous major and homozygous minor allele carriers (*p* < 0.05). Among those who were homozygous major (C/C), higher myristic acid levels were associated with reduced odds of AD incidence (OR 0.343 [95% CI 0.123;0.779], *p* = 0.022), whereas among those who were homozygous minor (T/T), higher odds of AD were observed (2.245 [1.127;5.582], *p* = 0.039). Heterozygous allele carriers presented no association of myristic acid with AD (0.912 [0.536;1.410], *p* = 0.701) (Figure 2).

## 4. Discussion

We analyzed the association of different fatty acids with the onset of AD up to one year of age in a large Indonesian birth cohort, testing both prospective and cross-sectional associations. Furthermore, we were interested in understanding the interaction of different fatty acids with variants of the *FADS1-3* gene cluster in this population.

The incidence of AD from birth to one year was 15.4%, slightly above the 12-month prevalence of 10.9–12.9% reported in a recent multinational epidemiologic study among children aged 6 months to 6 years in East Asian populations [30]. These values were based on data from Japan and Taiwan, whereas the prevalence among children aged 1–6 years in Malaysia has been reported at 13.4% [31]. Several characteristics differed significantly between subjects who developed AD and those who did not, including a higher proportion of parents with a history of atopy, and of parents with a higher level of education among AD subjects. Both these aspects have been previously reported to be associated with an increased risk of AD [32,33]. Results from a meta-analysis demonstrate that the effect of parental atopic history is even greater if coming from both parents, and also increases with the number of parental atopic diseases [32]. The most prominent explanation given for the higher AD prevalence rates observed in relation to higher socioeconomic position (as indicated by parental education in our study) is the “hygiene hypothesis” [34], or the extended “biodiversity hypothesis”, which assumes that a greater exposure to diverse microorganisms during pregnancy and early childhood in less affluent settings can be protective against allergies [35]. Others propose that subjects with a higher socioeconomic status are more likely to report allergic symptoms [36], but this does not apply in the present study, as all participants were followed up with the same regularity and AD was doctor-diagnosed. Subjects with AD in our study were also more frequently reported sick (cold, cough, and fever) in the first year of life, with an average of 24 days versus only 9 days in non-AD subjects. Patients with early AD onset typically experience immunological changes, resulting in more susceptibility to bacterial and viral infections [37]. There is also evidence of a correlation between respiratory viral infections and atopic diseases in children, both demonstrating similar pathophysiology in the production of serum specific IgE [38].

Correlations between fatty acids in different samples were the strongest between umbilical plasma and buccal cell samples collected at birth (P-0y and B-0y). LA and DHA presented the strongest positive correlations between their respective levels in the two samples (*ρ* > 0.5). Interestingly, the ARA/DGLA ratio reflecting Δ5 desaturase activity was strongly positively correlated; however, the GLA/LA and DHA/EPA ratios reflecting Δ6 desaturase activity were not. Correlations between the GPL-FA measured at birth and levels measured in buccal cheek cells at AD onset or at 1 year were weak, indicating a highly variable fatty acid profile within this brief period. Descriptive statistics demonstrated that the DHA/EPA ratio was much higher in P-0y than in B-0y and B-1y (23.2, 4.7, and 2.7, respectively), whereas EPA levels were similar across samples, thus possibly reflecting preferential materno-fetal DHA transfer across the placenta in P-0y [39].

We observed no significant prospective association of the GPL-FA measured at birth with an AD incidence up to 1 year of age. This is in contrast to previous findings, which indicated that lower omega-3 LC-PUFA concentrations in cord blood predicted AD among infants at 14 months [40]. However, in line with our results, a large prospective study observed no associations between cord blood LC-PUFA and AD at ages 2, 6, and 10 years [41]. The same study also assessed cross-sectional associations, and reported lower concentrations of n-3 LC-PUFA and a higher omega-6/omega-3 LC-PUFA ratio in children with AD at age 2 years [41]. Similarly, in our analyses, higher buccal cell (B-1y) ARA levels were cross-sectionally associated with higher odds of AD (OR = 2.021), while the opposite was observed for LA (OR = 0.567). As expected, a higher ARA/LA ratio in buccal cells indicative of increased Δ5 desaturase activity, was associated with AD. ARA serves as a precursor for the formation of eicosanoids, including prostaglandins (PG), prostacyclins (PGI), thromboxanes (TX) and leukotrienes (LT). These substances are known as pro-inflammatory mediators and T cell regulators. In AD pathophysiology, prostanoids and LT are implicated in causing pruritus, skin barrier disturbances, and type 2 immunity dysfunction [42]. On the other hand, our study did not indicate a protective effect of DHA; instead, we found a positive association of DHA with AD in B-1y (OR = 2.032). This may be a reflection of its high positive correlation with ARA (*ρ* = 0.73, Figure 1). It seems that the inflammation-resolving properties of DHA are outweighed by the increased availability of ARA, as the endogenous production of both LC-PUFA are intricately linked, given the competition for desaturase enzymes. We observed a positive association of oleic acid in B-1y samples (C18:1n-9) with AD (OR = 3.539). Within the B-1y sample, oleic acid was inversely correlated with all other GPL-FA (see supplementary Figure S1), which suggests that levels in buccal cells do not reflect endogenous oleic acid production, as this would also imply the increase in other Δ9 desaturase products (C16:1n7). Thus, our results suggest that higher exogenous oleic acid, possibly obtained through diet, may lead to an increased risk of AD. Studies in adults have reported a higher dietary intake of oleic acid to be positively associated with hay fever [43] and allergic sensitization [44]. However, an international study in children indicated the protective effects of olive oil consumption [45]. On the other hand, oleic acid is also known for its anti-inflammatory properties [46], and given the cross-sectional nature of our finding, reverse causation cannot be excluded, whereby infants presenting AD symptoms might be more frequently treated with oleic acid-rich products. Nevertheless, studies have demonstrated that topical use of oleic acid disrupts the skin barrier function [47], so a causal interpretation of the association is also plausible.

Our study demonstrates a significant interaction between rs174449 and myristic acid (C14:0) measured in buccal cells at birth (B-0y), which, to our knowledge, has not been reported previously. Stratified analyses indicated a positive association of myristic acid with AD in homozygous carriers of the minor allele (T/T), while major allele carriers (C/C) demonstrated an inverse association. Other correlated SNP presented at least nominally significant interactions with myristic acid (data not shown), suggesting that the observed interaction is unlikely a chance finding. Evidence indicating a role of myristic acid in the risk of AD is so far lacking. Myristic acid can be obtained from the diet, from sources such as dairy (constituting 8–14% of bovine milk [48]), but it is also a minor product of the de novo lipogenesis pathway [49]. Studies have demonstrated that myristic acid can regulate the activity of desaturases via N-terminal myristoylation [50]. Myristic acid has been reported to have a dose-dependent effect on Δ6 desaturase activity, thereby possibly regulating the LC-PUFA bioavailability [51]. These effects are suggested to occur by the myristoylation of the NADH-cytochrome b5 reductase, which is part of the whole desaturase complex. Myristic acid has, however, been found to specifically regulate the Δ4 desaturase activity, thus playing a role in the biosynthesis of ceramide and sphingolipid metabolism [52]. In this context, it makes sense that we would specifically observe an interaction with rs174449, as it is a variant of *FADS3*, and the FADS3 enzyme has been reported to act as a ceramide desaturase [53]. Specifically, FADS3 introduces a *cis* double bond in the C14 position of the long-chain base moiety of sphingosine-type ceramides, thereby producing sphingadiene-type ceramides. It is not long ago (in 2020) that this 4,14-sphingadiene base was identified in human skin for the first time [54]. The bent structure of the acyl chain due to the *cis* double bond is suggested to decrease the packing density of the lipid bilayer and weaken the lipid–lipid interaction [53]. While the relevance of this particular sphingoid base in AD still requires further study, altered sphingoid base profiles have been observed in relation to skin barrier abnormality in AD [55,56]. Our results point toward a possible role of FADS3 in AD etiology, with myristic acid representing a potential novel target for personalized preventive approaches. Furthermore, these findings may be of particular relevance for nanotechnology-based solutions, which offer promising new avenues for atopic dermatitis prevention and treatment, through improved skin bioavailability, targeted drug delivery at the inflammation site, and minimal side effects [57].

A major strength of the present study is the inclusion of a sizeable number of infants with a comprehensive assessment of the GPL-FA profile and doctor-diagnosed AD. We measured GPL-FA in buccal cheek cells, allowing for a less invasive sampling procedure and likely increased participation levels as a result. The further inclusion of umbilical artery plasma (P-0y) allowed for the comparison of the fatty acid levels across samples, while also highlighting clear sample-specific differences in association with AD. Furthermore, the study did not include participants whose mothers took omega-3 or omega-6 supplements, which would potentially dilute the effects resulting from the natural enzymatic pathway. To our knowledge, this is the first study in an Indonesian population to investigate the interaction of genetic variation, nutrition, and the incidence of AD in infants. Nevertheless, given the observational nature of the present study, we cannot infer causal effects, even though the prospective analyses are less likely to be affected by reverse causation.

## 5. Conclusions

We conclude that Indonesian infants developing AD within the first year of life have higher rates of Δ5 fatty acid desaturase activity, as reflected by higher ARA and lower LA levels as well as a higher ARA/LA ratio. Oleic acid levels are also positively associated with AD at the time of disease diagnosis. Fatty acid levels measured in cord blood or buccal cells at birth are not associated with AD incidence. However, genotype interaction analyses indicate an effect of myristic acid levels measured at birth on AD incidence, which is modified by the *FADS3* gene variant rs174449, and might offer new opportunities for precision prevention strategies.

## Figures and Tables

**Table 1:** Descriptive characteristics in the total population and stratified by AD presence.

	Total (N = 390)	AD = Yes (N = 60)	AD = No (N = 330)	*p*-Value
Sex (females)	191 (49)	29 (48)	162 (49)	1.000
Sex (males)	199 (51)	31 (52)	168 (51)	
Parental education (low)	79 (20)	6 (10)	73 (22)	**0.016**
Parental education (medium)	280 (72)	45 (75)	235 (71)	
Parental education (high)	31 (8)	9 (15)	22 (7)	
Breastfed (yes)	234 (60)	33 (55)	201 (61)	0.474
Breastfed (no)	156 (40)	27 (45)	129 (39)	
Paternal smoking (yes)	297 (76)	44 (73)	253 (77)	0.695
Paternal smoking (no)	93 (24)	16 (27)	77 (23)	
Atopic history (yes)	162 (42)	46 (77)	116 (35)	**<0.001**
Atopic history (no)	228 (58)	14 (23)	214 (65)	
Siblings (0)	121 (31)	20 (33)	101 (31)	0.916
Siblings (1)	141 (36)	21 (35)	120 (36)	
Siblings (2+)	128 (33)	19 (32)	109 (33)	
Frequency of illness (days)	11 (23)	23.5 (19)	9 (20)	**<0.001**

Values are presented as counts (%) for categorical variables and medians (interquartile range) for continuous variables. Differences between subjects with and without AD tested by Pearson’s chi-squared test and Wilcoxon Rank Sum test, for categorical and continuous variables, respectively. Significant differences marked in bold (*p* < 0.05).

**Table 2:** GPL-FA content in different samples.

	Umbilical Plasma (P-0y)		Buccal Cells Birth (B-0y)		Buccal Cells AD/1 Year (B-1y)	
Fatty Acids	n	wt%	n	wt%	n	wt%
C14:0	390	0.53 (0.12)	387	1.66 (0.91)	297	2.86 (2.06)
C16:0	390	29.8 (1.19)	386	17.6 (2.56)	299	21.3 (5.41)
C17:0	390	0.18 (0.06)	387	0.71 (0.26)	299	0.94 (0.50)
C18:0	390	14.8 (1.19)	387	18.1 (1.92)	299	12.8 (3.12)
C20:0	390	0.06 (0.05)		-		-
C14:1	390	0.03 (0.01)	386	0.07 (0.08)	294	0.07 (0.12)
C15:1	390	0.05 (0.02)	387	0.34 (0.18)	299	0.26 (0.23)
C16:1n-7	390	1.74 (0.50)	387	7.70 (1.94)	299	3.95 (1.87)
C18:1n-7	390	2.84 (0.48)	387	5.08 (0.92)	298	3.50 (1.20)
C18:1n-9	390	10.6 (1.36)	387	32.5 (3.29)	299	29.8 (3.93)
C20:1n-9	390	0.07 (0.02)	387	0.21 (0.16)	298	0.25 (0.18)
C20:3n-9	390	0.66 (0.44)		-		-
C16:1t	390	0.03 (0.03)	387	0.14 (0.10)	298	0.12 (0.10)
C18:1t	390	0.26 (0.16)	387	0.56 (0.66)	295	0.11 (0.11)
C18:2tt	390	0.08 (0.03)		-		-
C18:2n-6	388	8.61 (1.71)	387	5.96 (1.21)	299	17.2 (3.40)
C18:3n-6	390	0.15 (0.05)	387	0.22 (0.09)	298	0.11 (0.14)
C20:2n-6	390	0.48 (0.15)		-		-
C20:3n-6	390	5.64 (1.20)	387	1.69 (0.43)	297	0.69 (0.36)
C20:4n-6	390	15.5 (2.20)	387	3.91 (1.16)	299	1.72 (0.95)
C22:4n-6	390	0.63 (0.15)	387	0.40 (0.20)	298	0.25 (0.19)
C22:5n-6	390	1.25 (0.44)		-		-
C18:3n-3	388	0.05 (0.02)	387	0.32 (0.32)	299	0.60 (0.50)
C20:3n-3	390	0.13 (0.03)		-		-
C20:5n-3	389	0.10 (0.04)	386	0.12 (0.09)	299	0.13 (0.16)
C22:5n-3	390	0.21 (0.11)	387	0.27 (0.20)	299	0.30 (0.25)
C22:6n-3	390	5.03 (1.48)	387	1.25 (0.48)	299	0.82 (0.50)
Ratios	n	wt%/wt%	n	wt%/wt%	n	wt%/wt%
GLA/LA	388	0.02 (0.01)	387	0.04 (0.02)	298	0.01 (0.01)
ARA/DGLA	390	2.70 (0.91)	387	2.34 (0.65)	297	2.56 (1.43)
ARA/LA	388	1.79 (0.46)	387	0.66 (0.22)	299	0.10 (0.06)
DHA/DPA	390	23.2 (8.15)	383	4.76 (4.47)	299	2.71 (2.42)
DHA/ARA	390	0.32 (0.09)	387	0.32 (0.09)	299	0.48 (0.19)

Values presented as medians (interquartile range). wt%, percentage of total GPL-FA weight. GLA, γ -linolenic acid (C18:3n-6); LA, linoleic acid (C18:2n-6); ARA, arachidonic acid (C20:4n-6); DGLA, dihomo-γ -linolenic acid (C20:3n-6); DHA, docosahexaenoic acid (C22:6n-3); DPA, docosapentaenoic acid (C22:5n-3).

**Table 3:** Associations of GPL-FAs with AD.

		Umbilical Plasma (P-0y)			Buccal Cells Birth (B-0y)			Buccal cells AD/1 Year (B-1y)		
		OR	95% CI	*p*-Value	OR	95% CI	*p*-Value	OR	95% CI	*p*-Value
SFA	C14:0	1.220	0.872; 1.674	0.2283	0.971	0.704; 1.295	0.8480	0.929	0.598; 1.389	0.7292
	C16:0	0.775	0.522; 1.132	0.1954	0.898	0.620; 1.261	0.5517	0.533	0.314; 0.863	0.0141
	C17:0	1.217	0.779; 1.863	0.3743	1.062	0.786; 1.402	0.6784	0.652	0.399; 1.038	0.0784
	C18:0	1.083	0.731; 1.619	0.6920	0.871	0.639; 1.205	0.3889	1.138	0.777; 1.676	0.5088
	C20:0	1.051	0.870; 1.234	0.5729	-	-	-	-	-	-
MUFA	C14:1	1.228	0.874; 1.726	0.2356	0.839	0.590; 1.120	0.2800	0.667	0.438; 0.912	0.0295
	C15:1	1.079	0.869; 1.309	0.4608	1.223	0.918; 1.594	0.1493	0.875	0.671; 1.095	0.2801
Omega-7	C16:1n-7	1.251	0.831; 1.875	0.2777	1.031	0.693; 1.548	0.8806	1.058	0.691; 1.607	0.7917
	C18:1n-7	1.011	0.661; 1.534	0.9589	1.540	1.048; 2.295	0.0305	1.004	0.638; 1.589	0.9851
Omega-9	C18:1n-9	1.095	0.753; 1.582	0.6289	0.979	0.670; 1.452	0.9146	3.539	2.193; 5.988	**<0.001**
	C20:1n-9	0.923	0.608; 1.287	0.6651	0.93	0.729; 1.116	0.4957	1.297	0.945; 1.762	0.0989
	C20:3n-9	0.899	0.598; 1.303	0.5892	-	-	-	-	-	-
Trans	C16:1 t	0.984	0.594; 1.596	0.9486	0.966	0.763; 1.165	0.7393	0.761	0.507; 1.081	0.1563
	C18:1 t	0.714	0.461; 1.079	0.1192	1.041	0.841; 1.247	0.6782	1.229	0.925; 1.593	0.1289
	C18:2 tt	1.045	0.790; 1.355	0.7482	-	-	-	-	-	-
Omega-6	C18:2n-6	1.118	0.712; 1.760	0.6280	0.798	0.538; 1.145	0.2420	0.567	0.397; 0.800	**0.0015**
	C18:3n-6	1.282	0.849; 1.934	0.2345	0.941	0.742; 1.138	0.5680	0.884	0.648; 1.138	0.3849
	C20:2n-6	1.062	0.729; 1.525	0.7465	-	-	-	-	-	-
	C20:3n-6	0.992	0.657; 1.503	0.9701	1.015	0.675; 1.522	0.9427	1.072	0.910; 1.251	0.3765
	C20:4n-6	0.829	0.567; 1.217	0.3360	0.822	0.553; 1.213	0.3279	2.021	1.358; 3.072	**<0.001**
	C22:4n-6	1.298	0.889; 1.885	0.1723	0.922	0.729; 1.104	0.4172	1.275	0.945; 1.699	0.0998
	C22:5n-6	0.962	0.638; 1.434	0.8498	-	-	-	-	-	-
Omega-3	C18:3n-3	0.912	0.679; 1.175	0.5067	0.891	0.637; 1.155	0.4409	0.927	0.676; 1.226	0.6160
	C20:3n-3	1.063	0.702; 1.588	0.7686	-	-	-	-	-	-
	C20:5n-3	1.081	0.869; 1.321	0.4635	0.875	0.661; 1.096	0.2958	0.934	0.666; 1.243	0.6666
	C22:5n-3	1.452	1.030; 2.050	0.0326	0.954	0.669; 1.307	0.7789	0.905	0.633; 1.227	0.5516
	C22:6n-3	1.181	0.803; 1.734	0.3941	1.073	0.725; 1.582	0.7238	2.032	1.338; 3.131	**0.0010**
Ratios	GLA/LA	1.169	0.796; 1.691	0.4145	0.967	0.759; 1.184	0.7608	1.000	0.817; 1.152	0.9960
	ARA/DGLA	0.945	0.630; 1.387	0.7776	0.803	0.516; 1.229	0.3191	1.106	0.692; 1.763	0.6715
	ARA/LA	0.840	0.559; 1.233	0.3864	0.912	0.618; 1.337	0.6409	1.832	1.351; 2.595	**<0.001**
	DHA/DPA	0.591	0.371; 0.912	0.0215	1.085	0.768; 1.515	0.6368	1.472	0.975; 2.224	0.0646
	DHA/ARA	1.255	0.863; 1.820	0.2307	1.222	0.848; 1.760	0.2800	0.940	0.621; 1.356	0.7560

Associations tested by logistic regression adjusting for covariates (parental education, sex, family atopic history, exclusive breastfeeding for 4 months, paternal smoking, number of siblings, and frequency of illness). Significant associations after Bonferroni correction for multiple testing are marked in bold (*p* < 0.0019 for umbilical plasma, *p* < 0.0024 for buccal cells).

**Table 4:** Characteristics of analyzed SNPs.

SNP	Major/Minor Allele	MAF (%)	HWE *p*-Value
rs174448	T/C	47.7	0.684
rs174449	C/T	47.4	0.154
rs174455	C/T	45.5	0.414
rs174548	C/G	26.7	0.698
rs174556	A/G	26.7	0.698
rs174561	G/A	26.7	0.698
rs174570	T/C	23.2	0.568
rs174574	A/C	21.6	0.552
rs174575	C/G	29.3	0.902
rs174576	A/C	21.6	0.373
rs174578	A/T	21.6	0.373
rs174579	C/T	27.6	0.899
rs174602	C/T	41.2	0.531
rs2727271	T/A	49.5	0.684
rs3834458	DEL/T	21.9	0.460
rs498793	C/T	14.4	0.537
rs526126	C/G	22.5	0.080
rs968567	C/T	0.4	NA

MAF: minor allele frequency; HWE: Hardy-Weinberg equilibrium; NA: not available.

## Data Availability

Restrictions apply to the availability of these data. The datasets generated and/or analyzed during the current study are not publicly available due data protection but may be available on reasonable request and acceptance of a data transfer agreement from the legal department of the institution holding the data.

## Supplementary Materials

The following supporting information can be downloaded at: https://www.mdpi.com/article/10.3390/nu14214676/s1, Figure S1: Correlations of GPL-FA (wt%) within each sample; Table S1: Correlations between GPL-FA levels (wt%) in different samples.
