# Supplementary figures and images for: Novel Interactions of Myristic Acid and FADS3 Variants Predict Atopic Dermatitis among Indonesian Infants
Source: Nutrients. 2022 Nov 4;14(21):4676. doi: 10.3390/nu14214676 (PMC9653832; doi:10.3390/nu14214676)

Plasma (P-0y)

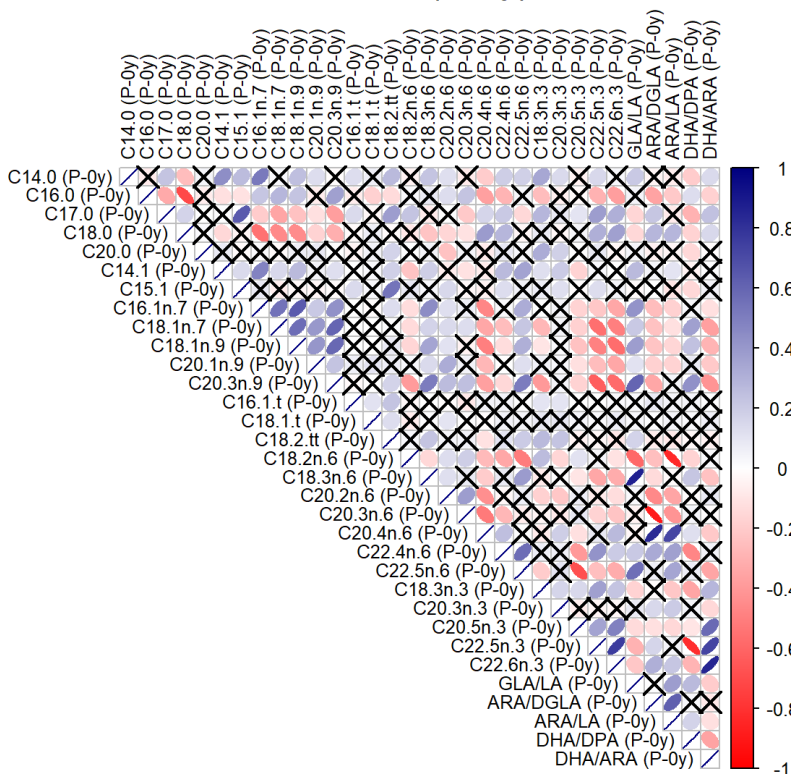

Buccal (B-0y)

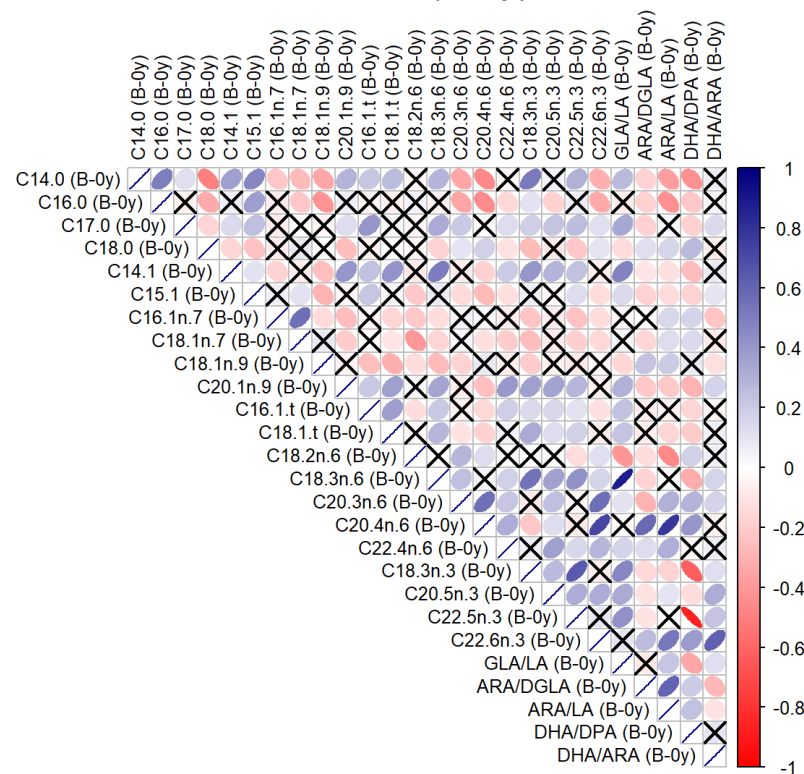

Buccal (B-1y)

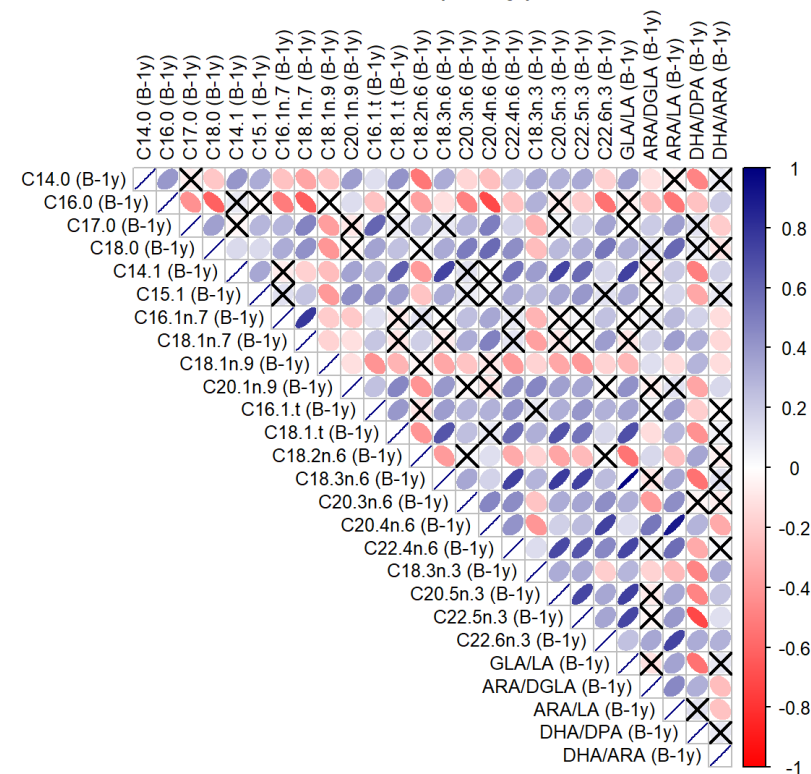

Supplement: Supplementary file 1 [file nutrients-14-04676-s001.zip › FigureS1_Supplement.pdf]
